# Supplementary material for: Peri-abortion contraceptive counseling: A systematic review of randomized controlled trials
Source: PLoS One. 2021 Dec 28;16(12):e0260794. doi: 10.1371/journal.pone.0260794 (PMC8714105; doi:10.1371/journal.pone.0260794)
Supplement: S1 Table — (DOCX) [file pone.0260794.s002.docx]

**S1 Table. Search terms.**

| Database or search engine | Search term | Number of results |
| --- | --- | --- |
| PubMed | (((“Counseling"[Mesh] OR Counsel*[tiab] OR "Directive Counseling"[Mesh])) AND ("Abortion, Spontaneous"[Mesh] OR miscarriag*[tw] OR "Early Pregnancy Loss"[tw] OR "Abortion, Induced"[Mesh] OR aborti*[tw] OR "Abortion, Therapeutic"[Mesh] OR "Abortion, Septic"[Mesh] OR "Abortion, Legal"[Mesh] OR "Abortion, Incomplete"[Mesh] OR "Abortion, Eugenic"[Mesh] OR "Abortion, Criminal"[Mesh] OR "Abortion Applicants"[Mesh])) AND ((randomized controlled trial[PT]) OR (controlled clinical trial[PT]) OR (clinical trial[PT]) OR (randomized[TIAB] OR randomised[TIAB]) OR (randomly[TIAB]) OR (trial[TIAB])) | 240 |
| SCOPUS | ( INDEXTERMS ( counseling ) OR TITLE-ABS-KEY ( counsel* ) OR INDEXTERMS ( directive AND counseling ) ) AND ( INDEXTERMS ( spontaneous AND abortion ) OR TITLE-ABS ( miscarriag* ) OR TITLE-ABS-KEY ( early AND pregnancy AND loss ) OR INDEXTERMS ( induced AND abortion ) OR TITLE-ABS-KEY ( aborti* ) OR INDEXTERMS ( therapeutic AND abortion ) OR INDEXTERMS ( septic AND abortion ) OR INDEXTERMS ( legal AND abortion ) OR INDEXTERMS ( incomplete AND abortion ) OR INDEXTERMS ( eugenic AND abortion ) OR INDEXTERMS ( criminal AND abortion ) OR INDEXTERMS ( abortion AND applicants ) ) AND ( TITLE-ABS-KEY ( "randomized controlled trial" ) OR TITLE-ABS-KEY ( "controlled clinical trial" ) OR TITLE-ABS-KEY ( "clinical trial" ) OR TITLE-ABS-KEY ( randomized ) OR TITLE-ABS-KEY ( randomised ) OR TITLE-ABS-KEY ( randomly ) OR TITLE-ABS-KEY ( trial ) ) | 563 |
| CENTRAL | abortion AND counseling AND trial | 5 |
| Google Scholar | abortion counseling trial | First 100 results |
| Search date: May 2020 | | |
