# Supplementary material for: Peri-abortion contraceptive counseling: A systematic review of randomized controlled trials
Source: PLoS One. 2021 Dec 28;16(12):e0260794. doi: 10.1371/journal.pone.0260794 (PMC8714105; doi:10.1371/journal.pone.0260794)
Supplement: S2 Table — (DOCX) [file pone.0260794.s003.docx]

**S2 Table. List of studies that were assessed in full-text and excluded.**

| **Author** | **Year** | **Title** | **Exclusion reason** |
| --- | --- | --- | --- |
| Adewole | 2002 | Contraceptive usage among abortion seekers in Nigeria | Non-randomized controlled trial |
| Banerjee | 2015 | Associations Between Abortion Services and Acceptance of Postabortion Contraception in Six Indian States | Non-randomized controlled trial |
| Bracken | 1973 | Abortion counseling: An experimental study of three techniques | No outcome of interest |
| Brown | 2019 | Shared negative experiences of long-acting reversible contraception and their influence on contraceptive decision-making: a multi-methods study | Non-randomized controlled trial |
| Cameron | 2014 | Postabortal and postpartum contraception | Non-randomized controlled trial |
| DePiñeres | 2014 | Randomized controlled trial of contraceptive use at 3 and 6 months postabortion | Full-text was not available |
| El-Tagy | 2003 | Safety and acceptability of post-abortal IUD insertion and the importance of counseling | Non-randomized controlled trial |
| Emtell | 2021 | Increasing uptake of long-acting reversible contraception with structured contraceptive counselling: cluster randomised controlled trial (the LOWE trial) | No outcome of interest |
| Ferreira | 2010 | Choices on contraceptive methods in post-abortion family planning clinic in the northeast Brazil | Non-randomized controlled trial |
| Gemzell-Danielsson | 2014 | Contraception following abortion and the treatment of incomplete abortion | Non-randomized controlled trial |
| Gerdts | 2020 | Effect of a smartphone intervention on self-managed medication abortion experiences among safe-abortion hotline clients in Indonesia: A randomized controlled trial | No outcome of interest |
| Grace | 2015 | Author's reply re: The impact of supportive counseling on women's psychological wellbeing after miscarriage - A randomised controlled trial | Letter to the editor |
| Grosu | 2015 | Re: The impact of supportive counseling on women's pyschological wellbeing after miscarriage – a randomised controlled trial | Letter to the editor |
| Gunyeli | 2012 | Contraceptive counseling in Turkey after induced abortion | Non-randomized controlled trial |
| Harper | 2015 | Reductions in pregnancy rates in the USA with long-acting reversible contraception: A cluster randomised trial | No outcome of interest |
| Hill | 2020 | A mobile phone-based support intervention to increase use of postabortion family planning in Cambodia: Cost-effectiveness evaluation | No outcome of interest |
| Hofmeyr | 2016 | Effects of the copper intrauterine device versus injectable progestin contraception on pregnancy rates and method discontinuation among women attending termination of pregnancy services in South Africa: A pragmatic randomized controlled trial | No outcome of interest |
| Kong | 2014 | The impact of supportive counseling on women's psychological wellbeing after miscarriage--a randomised controlled trial | No outcome of interest |
| Langston | 2014 | Immediate postabortion access to IUDs, implants and DMPA reduces repeat pregnancy within 1 year in a New York City practice | Non-randomized controlled trial |
| Makenzius | 2018 | Contraceptive uptake in post abortion care-Secondary outcomes from a randomised controlled trial, Kisumu, Kenya | Non-randomized controlled trial |
| Mandira | 2016 | Does mode of follow-up influence contraceptive use after medical abortion in a low-resource setting? Secondary outcome analysis of a non-inferiority randomized controlled trial | No outcome of interest |
| Matulich | 2014 | Understanding women's desires for contraceptive counseling at the time of first-trimester surgical abortion | Non-randomized controlled trial |
| Nagai | 2019 | Opportunities lost: Barriers to increasing the use of effective contraception in the Philippines | Non-randomized controlled trial |
| Nikcević | 2007 | The influence of medical and psychological interventions on women's distress after miscarriage | No outcome of interest |
| Purcell | 2016 | Contraceptive care at the time of medical abortion: Experiences of women and health professionals in a hospital or community sexual and reproductive health context | Non-randomized controlled trial |
| Rasch | 2004 | Acceptance of contraceptives among women who had an unsafe abortion in Dar es Salaam | Non-randomized controlled trial |
| Rasch | 2007 | Acceptance and use of the female condom among women with incomplete abortion in rural Tanzania | Non-randomized controlled trial |
| Rehnström | 2020 | Contraceptive uptake among post-abortion care-seeking women with unplanned or planned pregnancy in western Kenya | Non-randomized controlled trial |
| Reiss | 2017 | Using automated voice messages linked to telephone counseling to increase post-menstrual regulation contraceptive uptake and continuation in Bangladesh: study protocol for a randomised controlled trial | Non-randomized controlled trial |
| Rocca | 2018 | Contraception after medication abortion in the United States: results from a cluster randomized trial | No outcome of interest |
| Rocca | 2016 | Funding policies and postabortion long-acting reversible contraception: results from a cluster randomized trial | No outcome of interest |
| Roe | 2020 | Pregnancy intentions and contraceptive uptake after miscarriage | Non-randomized controlled trial |
| Roe | 2018 | Advance notice of contraceptive availability at surgical abortion: A pilot randomised controlled trial | No outcome of interest |
| Rose | 2010 | Uptake and adherence to long-acting reversible contraception post-abortion | Non-randomized controlled trial |
| Sääv | 2012 | Early versus delayed insertion of intrauterine contraception after medical abortion - a randomized controlled trial | No outcome of interest |
| Samuel | 2016 | Strengthening Postabortion Family Planning Services in Ethiopia: Expanding Contraceptive Choice and Improving Access to Long-Acting Reversible Contraception | Non-randomized controlled trial |
| Sapkota | 2017 | The Impact of Balanced Counseling on Contraceptive Method Choice and Determinants of Long Acting and Reversible Contraceptive Continuation in Nepal | Non-randomized controlled trial |
| Saravelos | 2011 | The importance of preconception counseling and early pregnancy monitoring | Non-randomized controlled trial |
| Sedlecky | 2016 | Contraception for adolescents after abortion | Non-randomized controlled trial |
| Shimoni | 2011 | Timing of Copper Intrauterine Device Insertion After Medical Abortion: A Randomized Controlled Trial | No outcome of interest |
| Smith | 2017 | Process evaluation of a mobile phone-based intervention to support post-abortion contraception in Cambodia | Non-randomized controlled trial |
| Smith | 2019 | Characteristics and contraceptive outcomes of women seeking medical or surgical abortion in reproductive health clinics in Cambodia | Non-randomized controlled trial |
| Smith | 2013 | MObile Technology for Improved Family Planning Services (MOTIF): Study protocol for a randomised controlled trial | Non-randomized controlled trial |
| Smith | 2017 | Women's views and experiences of a mobile phone-based intervention to support post-abortion contraception in Cambodia | No outcome of interest |
| Sonalkar | 2017 | A randomized pilot evaluation of individual-level abortion stigma resulting from Pennsylvania mandated abortion counseling | No outcome of interest |
| Sonalkar | 2018 | Postabortion long-acting reversible contraception desire in women counselled using Bedsider.org versus standard counseling: a randomised trial | Letter to the editor |
| Sultana | 2019 | Induced abortion and sub-sequent prevalence of contraceptive type | Non-randomized controlled trial |
| Thompson | 2018 | Training contraceptive providers to offer intrauterine devices and implants in contraceptive care: a cluster randomized trial | No outcome of interest |
| Voetagbe | 2010 | Midwifery tutors' capacity and willingness to teach contraception, post-abortion care, and legal pregnancy termination in Ghana | Non-randomized controlled trial |
| Whitaker | 2015 | Motivational interviewing to improve postabortion contraceptive uptake by young women: development and feasibility of a counseling intervention | Non-randomized controlled trial |
| Whitaker | 2014 | Motivational interviewing to improve postabortion uptake of long-acting reversible contraception by young women: a randomized controlled trial | Full-text was not available |
