# Supplementary material for: Peri-abortion contraceptive counseling: A systematic review of randomized controlled trials
Source: PLoS One. 2021 Dec 28;16(12):e0260794. doi: 10.1371/journal.pone.0260794 (PMC8714105; doi:10.1371/journal.pone.0260794)
Supplement: S3 Table — (DOCX) [file pone.0260794.s004.docx]

**S3 Table. Characteristics of the studies, in extenso.**

| Author | Date and setting | Randomized Unit | Inpatient/ Outpatient | Age | Participants | Other inclusion and exclusion criteria | Where abortion was performed | Where they were recruited | Marital Status | History of Previous Abortion | Number of patients | Counseling interventions given to both groups | Difference between intervention and Control | Follow up |
| --- | --- | --- | --- | --- | --- | --- | --- | --- | --- | --- | --- | --- | --- | --- |
| Bender 2004, Iceland | 1999-2000 | Women | Inpatient | 24.4 | Women who requested first-trimester pregnancy termination in the abortion clinic at the Landspitali University Hospital | Women who did not speak Icelandic or were under great emotional strain were excluded | Landspitali University | Landspitali University |  | 57 (24.78) | 276 (148,128) | None | I: Routine care by a social worker and physician was given once + Pre abortion specialized counseling by a specially trained family planning nurse given twice  C: Routine care from a nurse/midwife without special training in contraceptive counseling given once. | 6 months |
| Schunmann 2006, United Kingdom | 2002 | Weeks | Inpatient |  | Women presenting at the abortion clinic without obstetric indication for pregnancy termination. | Women undergoing termination for fetal abnormality, those whose command of English was poor and a few women thought by the nursing staff who were distributing the questionnaire to be too distressed to be approached, were excluded from the study | Abortion Clinic at the Royal Infirmary of Edinburgh | Abortion Clinic at the Royal Infirmary of Edinburgh |  | Not evaluated | 377(199.178) | SC brief discussion abut contraception and assessment at the outpatient clinic | Expert advice by a doctor + Enhanced contraception of 3-month provision pills, implants, or IUD/IUS. | 16 weeks |
| Nobili 2007, Italy | 2004 | Women | Outpatient |  | Women who requested termination of pregnancy | psychiatric pathology, fetal malformations, or insufficient address information for follow-up. Foreign women were also excluded from the study, due to the language and communication difficulties involved | San Paolo Hospital of Milan | San Paolo Hospital of Milan |  | Not evaluated | 43 (22, 21) |  | 30 min personalized contraceptive counseling consisting of a patient centered interview, information and education, the election of contraceptive and checking understanding of contraceptive use before the Termination of Pregnancy by a psychologist and gynecologist | 3 months |
| Zhu 2009 China | 2006 | Hospital | Outpatient |  | Women seeking abortion | Women, who were less than 25 years old and in the first trimester of pregnancy | Beijing, Sahnghai and Zhengzhou Abortion clinic | Beijing, Sahnghai and Zhengzhou Abortion clinic |  | 853 (36.64) | 2336(1147, 1189) |  | A)Essential package based on the provision of information and referral to women to existing family planning services B) Comprehensive package consisted of individual and group education and male involvement in both, free provision of contraception and referral of women to existing family planning service. | 6 months |
| Langston 2010 USA | 2008-2009 | Women | Inpatient | 26.2 +- 6.2 | Women seeking a first-trimester abortion for a spontaneous or induced abortion | No desire to become pregnant right away, fluency in Spanish or English, and access to a telephone | family planning referral clinic to a private practice setting | family planning referral clinic to a private practice setting |  | 115 (51.8) | 222 | SC consisted of providing abortion and contraceptive counseling performed by a physician with content and duration left to their discretion | Standardized Structured Counseling using visual and audio material and contraception provision | 3 months |
| Carneiro 2011 Brazil | 2008 | Women | Outpatient | 26.8 +_ 6.8 | Women who had undergone any kind of abortion | Except for gestational trophoblastic disease, benign and malignant tumors of the uterus and uterine malformations | Five public maternities in Recife | Five public maternities in Recife | Cohabitant: 184/246 | 68 (27.64) | 426 | SC: group counseling educational counseling and gynecology visit | Individually Personalized three-stage 30 minutes counseling by two trained providers (education and information, guided information and free provision of chosen contraceptive and verification of their understanding of their use) | 6 months |
| Smith 2015 Cambodia | 2013 | Weeks | Outpatient |  | Women who sought induced abortion and has a mobile phone | reported not wanting to become pregnant and were willing to receive automated voice messages about contraception | Four Marie Stopes International clinics in Cambodia | Four Marie Stopes International clinics in Cambodia |  | 201 (40.20) | 500 | Standard care | Mobile phone-based intervention consisting of six automated interactive voice messages at the time of their preference during 3 months | 12 months |
| Olavarrieta 2015 Mexico | 2013 | Health care provider | Inpatient | 26 +- 6.2 | Women looking for medical abortion | Previous medical conditions, allergy to mifepristone or misoprostol, or if they already had previously received medical abortion as part of the Mexico City Legal abortion program. | Two Mexico City Ministry of Health abortion clinics and one hospital | Two Mexico City Ministry of Health abortion clinics and one hospital |  | Not evaluated | 1017 |  | I: Contraceptive method counseling provided by a Nurse C: Contraceptive method counseling provided by Physician | 15 days |
| Davidson 2015 USA | 2013 | Women | Inpatient | 23.35 +- 3.1 | Women presenting for a surgical abortion | nonviable or anomalous pregnancy, pregnancy as a result of sexual assault, or an inability to speak, read, and/or write in English | Freestanding clinic | Freestanding clinic |  | 97 (50.52) | 192 | SC consisting of contraception and abortion counseling by clinic staff before abortion | I: Long-acting contraception informative video  C: Stress management video | None |
| Whitaker 2016 USA | 2013 | Women | Outpatient | 22.8 +- 3.8 | Women who sought an abortion from 15 to 29 years old | Women requesting abortion for fetal or maternal medical indications with a pregnancy resulting from sexual assault or desire for repeat pregnancy in six months. | Urban academic center | Urban academic center" | Cohabitant 9 (15%) | 24 (40.00) | 60(29,31) | Standard care | Seven steps motivational Interview provided by physician and physician or social worker | 3 Month |
| Makenzius 2017 USA | 2013-2016 | Healthcare provider | Inpatient | 25 +- 5.7 | Women with signs of incomplete abortion based on clinical assessment but without the use of ultrasound | complete abortion, a uterine size estimated over 12 weeks of gestation, suspected ectopic pregnancy, unstable hemodynamic status, signs of pelvic infection or sepsis, and known allergy to misoprostol | Hospital | Hospital |  | Not evaluated | 409 |  | I: Contraceptive counseling provided by a Midwife C: Contraceptive counseling provided by Physician | 10 days |
