# Supplementary material for: Peri-abortion contraceptive counseling: A systematic review of randomized controlled trials
Source: PLoS One. 2021 Dec 28;16(12):e0260794. doi: 10.1371/journal.pone.0260794 (PMC8714105; doi:10.1371/journal.pone.0260794)
Supplement: S4 Table — (DOCX) [file pone.0260794.s005.docx]

**S4 Table. Definition of the outcomes assessed in the meta-analyses in each study.**

| Outcome | Contraceptive methods | Bender 2004 (Islandia) | Schunmann 2006 (United Kingdom) | Nobili 2007 (Italy) | Zhu 2009 (China) | Langston 2010 (USA) | Carneiro 2011 (Brazil) | Smith 2015 (Cambodia) | Davidson 2015 (USA) | Whitaker 2016 (USA) |
| --- | --- | --- | --- | --- | --- | --- | --- | --- | --- | --- |
| Use of an effective contraceptive | Oral contraceptive | x | x | x | x | x | x | x |  | x |
|  | Intrauterine device | x | x | x | x | x | x | x |  | x |
|  | Implant |  | x |  | x | x |  | x |  |  |
|  | Hormonal injection | x | x | x |  |  | x | x |  | x |
|  | Sterilization/vasectomy |  | x |  |  | x |  | x |  |  |
|  | Condom | x | x | x | x |  | x |  |  |  |
|  | Vaginal ring |  |  | x |  | x |  |  |  |  |
|  | Contraceptive patch |  |  | x |  | x |  |  |  |  |
| Use of long-acting reversible contraceptives | Definition to LARC | Not defined | Not defined |  |  |  |  |  |  | Defined |
|  | IUD | x | x |  |  |  |  |  |  |  |
|  | IUS |  | x |  |  |  |  |  |  | x |
|  | Implant |  | x |  |  |  |  |  |  | x |
| Uptake of an effective contraceptive method | Oral contraceptives |  | x |  |  | x | x |  | x | x |
|  | Patch |  |  |  |  | x |  |  | x |  |
|  | Ring |  |  |  |  | x |  |  |  |  |
|  | Monthly injectable |  | x |  |  | x | x |  |  |  |
|  | Quarterly injectable |  | x |  |  | x | x |  | x | x |
|  | Condom |  | x |  |  |  | x |  |  |  |
|  | Implant |  |  |  |  | x |  |  | x | x |
|  | IUD/IUS |  | x |  |  | x | x |  | x | x |
|  | Sterilization |  | x |  |  | x |  |  |  |  |
| Occurrence of an obstetric event | Pregnancy |  |  |  | x |  | x | x |  |  |
|  | Induced abortion |  |  |  | x |  |  | x |  |  |
