# Supplementary material for: Peri-abortion contraceptive counseling: A systematic review of randomized controlled trials
Source: PLoS One. 2021 Dec 28;16(12):e0260794. doi: 10.1371/journal.pone.0260794 (PMC8714105; doi:10.1371/journal.pone.0260794)
Supplement: S5 Table — (DOCX) [file pone.0260794.s006.docx]

**S5 Table. Detail of the interventions received in Bender´s study.**

| **TIDieR** | **INTERVENTION** | **CONTROL** |
| --- | --- | --- |
|  | **Bender 2004** | |
| MATERIALS | Leaflets | Leaflets |
| PROCEDURES | 1. Routine counseling. 2. Differentiated intervention: “The intervention interview focused on their contraceptive pattern through the years. Their contraceptive history was plotted on a line, where age at initial use of contraceptives was on the left and their present age on the right. The types of contraceptives used were entered along the timeline as well as childbirths. The nonuse of contraception was explored with the woman as were her future plans to use contraception. The focus on previous, present and prospective contraceptive use was considered to raise women’s awareness towards contraception (…) . Every woman received information about return of fertility after the abortion and general and emergency contraception. They were all provided with written information about the contraceptive methods that they were planning to use” 3. Interview: "The intervention interview included background data, general use of contraceptive methods, reasons for nonuse, use of contraceptives at the time of conception and future planning of contraceptive use"  4. Evaluation time: "The follow-up interview of the intervention group was focused on the contraceptive method they had started to use, the type and reasons for choice of method, method compliance and method time plan" | 1. Routine counseling. 2. Interview: "The intervention interview included background data, general use of contraceptive methods, reasons for nonuse, use of contraceptives at the time of conception and future planning of contraceptive use"  3. Evaluation time: "Focused on the contraceptive method they had started to use, the type and reasons for choice of method, method compliance and method time plan" |
| WHO PROVIDED | Routine Care: social worker and physician Pre abortion counselling: specially trained family-planning nurse | Routine care from a nurse or a midwife without special training |
| HOW | Not specified | Not specified |
| WHERE | Abortion clinic at Landspitali University Hospital | Abortion clinic at Landspitali University Hospital |
| WHEN | Pre-abortion | Post-abortion |
| HOW MUCH | Twice | once |
| TAILORING | Personalized | None |
| MODIFICATIONS | None | None |
| Adherence evaluation | No | No |
