# Supplementary material for: Peri-abortion contraceptive counseling: A systematic review of randomized controlled trials
Source: PLoS One. 2021 Dec 28;16(12):e0260794. doi: 10.1371/journal.pone.0260794 (PMC8714105; doi:10.1371/journal.pone.0260794)
Supplement: S6 Table — (DOCX) [file pone.0260794.s007.docx]

**S6 Table. Detail of the interventions received in Schunmann ´s study.**

| **TIDieR** | **INTERVENTION** | **CONTROL** |
| --- | --- | --- |
|  | **Schunmann 2006** | |
| MATERIALS | Contraception provision: oral contraception for 3 months of any brand and implants | Contraception provision: oral contraception only one packet of combined or progestogen-only pill, and only one brand of each was available and contraceptive implants were not provided |
| PROCEDURES | 1. Standard care “Women consenting to take part in the study and admitted for abortion during an intervention week underwent the same procedures, including brief discussion about future contraception during the initial consultation and assessment in the outpatient clinic” and “offered expert advice doctor with specialist training in contraception and enhanced provision of contraception” 2. Enhanced contraception: "The preferred method of contraception for use after abortion was discussed, and a supply, sufficient for 3 months, of the chosen method (and brand if relevant) was dispensed. (…), or implants inserted, before discharge from hospital. An IUD/IUS was inserted in the operating theatre following STOP but not immediately after MTOP; rather, firm arrangements were made for insertion at a local family planning clinic (FPC) after routine hospital follow-up at 2 weeks". | Standard Care: "Standard care comprised of a brief discussion about contraception at the initial outpatient consultation before the abortion procedure was arranged and further discussion by the nursing staff before the women left hospital after the abortion and Contraception Provision" |
| WHO PROVIDED | Standard Care: physician | Pre abortion counselling: not specified Post abortion counselling: nurse |
| HOW | Not specified | Not specified |
| WHERE | Abortion clinic at the royal Infirmary of Edimburgh | Abortion clinic at the royal Infirmary of Edimburgh |
| WHEN | Pre or post-abortion | Pre-abortion and post-abortion |
| HOW MUCH | Once, 15 to 20 min | Once |
| TAILORING | No specified | No specified |
| MODIFICATIONS | A few women undergoing surgical abortion were interviewed post procedure if there was no time to see them before the procedure. | No |
| Adherence evaluation | No | No |
