# Supplementary material for: Peri-abortion contraceptive counseling: A systematic review of randomized controlled trials
Source: PLoS One. 2021 Dec 28;16(12):e0260794. doi: 10.1371/journal.pone.0260794 (PMC8714105; doi:10.1371/journal.pone.0260794)
Supplement: S7 Table — (DOCX) [file pone.0260794.s008.docx]

**S7 Table. Detail of the interventions received in Nobili´s study.**

| **TIDieR** | **INTERVENTION** | **CONTROL** |
| --- | --- | --- |
|  | **Nobili 2007** | |
| MATERIALS | None | None |
| PROCEDURES | 1. Personalized contraceptive counseling intervention of 30 minutes. It was conducted by a psychologist and a gynecologist and consisted of three phases: a. Patient-centered semi-structured interview (10 min): This first part, conducted by the psychologist, aimed to explore the woman’s ‘‘agenda’’ regarding contraception, including her barriers to use, her perceptions of risk and her past and present experiences of contraception. b. Offer of information and education (15 min): This second phase was conducted by a gynecologist who presented the advantages and disadvantages of the available effective contraceptive methods (condom, pill, IUD, vaginal ring, contraceptive patch) as well as explaining how to use them. Information about how to obtain and use emergency contraception was also presented here. c. Choosing the contraceptive method and checking understanding (5 min): In the third phase, conducted by the psychologist, the woman decided which method was the most appropriate for her as a result of the exploration of her agenda and the information obtained in the previous stage. The psychologist and the gynecologist could also answer any remaining doubts or questions, checking the woman’s understanding and satisfaction with the counseling intervention. 2. Evaluation time: Telephone Questionnaire 1month after TOP and Telephone Questionnaire 3 months after TOP | 1. Standard Care This consists in encouraging women to consult the community health centers after TOP. 2. Evaluation time: Telephone Questionnaire 1month after TOP and Telephone Questionnaire 3 months after TOP |
| WHO PROVIDED | Pre abortion counselling: psychologist and gynecologist | Standard care: not specified |
| HOW | No specified | No specified |
| WHERE | San Paolo Hospital of Milan | San Paolo Hospital of Milan |
| WHEN | Pre-abortion | Post-abortion |
| HOW MUCH | Once 30 minutes | No detail |
| TAILORING | Personalized | No detail |
| MODIFICATIONS | No | No |
| Adherence evaluation | No | No |
