# Supplementary material for: Peri-abortion contraceptive counseling: A systematic review of randomized controlled trials
Source: PLoS One. 2021 Dec 28;16(12):e0260794. doi: 10.1371/journal.pone.0260794 (PMC8714105; doi:10.1371/journal.pone.0260794)
Supplement: S8 Table — (DOCX) [file pone.0260794.s009.docx]

**S8 Table. Detail of the interventions received in Zhu’s study.**

| **TIDieR** | **INTERVENTION** | **CONTROL** |
| --- | --- | --- |
|  | **Zhu 2009** | |
| MATERIALS | Contraception provision: free condoms, oral contraceptives [OCs], intrauterine devices [IUDs], implants | NONE |
| PROCEDURES | Group education and individual counselling for men and women | Group education and referral to a Family Planning service |
| WHO PROVIDED | Comprehensive package not specified | Essential package: not specified. |
| HOW | Group education and face-to-face counselling | Group education |
| WHERE | Abortion clinic, Sahnghai and Zhengzhou , Beijing | Beijing, Sahnghai and Zhengzhou ,Abortion clinic |
| WHEN | Pot-abortion | Post-abortion |
| HOW MUCH | Not specified | Not specified |
| TAILORING | Not specified | Not specified |
| MODIFICATIONS | No | No |
| Adherence evaluation | No | No |
