# Supplementary material for: Peri-abortion contraceptive counseling: A systematic review of randomized controlled trials
Source: PLoS One. 2021 Dec 28;16(12):e0260794. doi: 10.1371/journal.pone.0260794 (PMC8714105; doi:10.1371/journal.pone.0260794)
Supplement: S9 Table — (DOCX) [file pone.0260794.s010.docx]

**S9 Table. Detail of the interventions received in Lagnston´s study.**

| **TIDieR** | **INTERVENTION** | **CONTROL** |
| --- | --- | --- |
|  | **Lagnston 2010** | |
| MATERIALS | Procedure materials: visual and audio components  Contraception provision: The IUDs and implants were donated and available at no cost to all clinic patients, those who chose pill or ring received either a prescription or a 1-month supply and prescription. All participants received condoms with handouts on emergency contraception and condom use. | Procedure materials: visual and audio components |
| PROCEDURES | 1. Usual Care: Single physician performing the medical history, physical exam, ultrasound, obtaining informed consent for the suction aspiration procedure, and carrying out this procedure for each patient. This visit required about one hour to complete. Contraceptive counseling was routinely offered by the physician as well and was embedded in the visit. As part of usual care, the content and duration of contraceptive counseling performed by the provider was left to their discretion.  2. Intervention: Trained research coordinator reading and displaying a contraceptive flipchart in its entirety to the participant in a private office with samples of each method available for patients to see and touch. The counseling was structured in that the format included visual and audio components allowing the participant to both visualize and hear the information. The counseling was standardized in that the same information was presented every time the counseling was performed. Participants were encouraged to ask questions and to write down questions for their physician on supplied note cards. The research assistants were trained to answer questions using only the information from the flipchart. If a question was not able to be answered by the information on the flipchart, the research assistant was instructed to request the participant ask her provider this question during usual care."  3. About contraceptive methods: Available to participants immediately following their procedure included intrauterine devices (IUDs), implants, injections, rings, and pills. The IUDs and implants were donated and available at no cost to all clinic patients. All participants had either New York State Medicaid coverage for prescription contraceptives or access to additional free supplies at a safety net clinic, so all contraceptives offered were available free of charge. The patch was available by prescription only and sterilization by referral only. Those who chose pill or ring received either a prescription or a 1-month supply and prescription. All participants received condoms with handouts on emergency contraception and condom use. 4. Evaluation time: "After each enrollment day, we reviewed charts to confirm that a procedure was performed and to identify the contraceptive method chosen as well as whether initiation was immediate or delayed. Coordinators called participants 3 months after enrollment to assess contraceptive use. A subset of patients received 6 months follow-up phone calls. Initial analysis of the first 101 participants to complete both 3- and 6-months data found no significant differences, so 6 months follow-up was stopped to focus on maximizing 3 months follow-up". | Usual Care: Usual care consisted of a single physician performing the medical history, physical exam, ultrasound, obtaining informed consent for the suction aspiration procedure, and carrying out this procedure for each patient. This visit required about one hour to complete. Contraceptive counseling was routinely offered by the physician as well and was embedded in the visit. As part of usual care, the content and duration of contraceptive counseling performed by the provider was left to their discretion. |
| WHO PROVIDED | Pre abortion counselling: research coordinator Standard care: physician | Usual care: physician |
| HOW | Face to face | Face-to-face |
| WHERE | Family planning referral clinic to a private practice setting in New York City | Family planning referral clinic to a private practice setting in New York City |
| WHEN | Pre-abortion | Post-abortion |
| HOW MUCH | Once, Intervention length Not specified and Standard Care length left to provider discretion | Once and Standard Care length left to provider discretion |
| TAILORING | We did not intend the intervention to provide tailored counseling, though that is one of the common uses of the Decision-Making Tool (DMT). | None |
| MODIFICATIONS | No | No |
| Adherence evaluation | No | No |
