# Supplementary material for: Peri-abortion contraceptive counseling: A systematic review of randomized controlled trials
Source: PLoS One. 2021 Dec 28;16(12):e0260794. doi: 10.1371/journal.pone.0260794 (PMC8714105; doi:10.1371/journal.pone.0260794)
Supplement: S10 Table — (DOCX) [file pone.0260794.s011.docx]

**S10 Table. Detail of the interventions received in Carneiro´s study.**

| **TIDieR** | **INTERVENTION** | **CONTROL** |
| --- | --- | --- |
|  | **Carneiro 2011** | |
| MATERIALS | Not detailed | none |
| PROCEDURES | 1.Intervention: personalized counseling A. Education and information: This constitutes individual counseling to inform about fertility after abortion, ideal birth interval for women's reproductive health, advantages, and disadvantages of all types of contraceptive methods available and how to use them. Information about emergency contraception also was provided (15 min). B. Provider-guided information based on the three points: This includes plan to use contraceptive methods, experience with last method used and expectations, beliefs and myths related to contraception (10 min). C. Free provision of chosen contraceptive method and verification of their understanding of its use (5 min). 2. Contraceptive method was given at no cost" | Standard care: 30- to 40-min educational group counseling in family planning and discussion about contraceptive methods and their side effects, followed by a visit to the gynecologist. |
| WHO PROVIDED | Post-abortion counselling: Two trained providers | Standard Care: specialized nursing staff in family planning followed by a visit to the gynecologist |
| HOW | Facet to face | Group counseling from 20-25 women |
| WHERE | Family planning clinic at Instituto de Medicina Integral Prof. Fernando Figueira (IMIP) | Family planning clinic at Instituto de Medicina Integral Prof. Fernando Figueira (IMIP) |
| WHEN | Postabortion | Postabortion |
| HOW MUCH | Once for 30 minutes | Once, 30- to 40-min |
| TAILORING | Personalized regarding the individual's contraceptive history and needs. | No |
| MODIFICATIONS | No | No |
| Adherence evaluation | No | No |
