# Supplementary material for: Peri-abortion contraceptive counseling: A systematic review of randomized controlled trials
Source: PLoS One. 2021 Dec 28;16(12):e0260794. doi: 10.1371/journal.pone.0260794 (PMC8714105; doi:10.1371/journal.pone.0260794)
Supplement: S11 Table — (DOCX) [file pone.0260794.s012.docx]

**S11 Table. Detail of the interventions received in Davidson´s study.**

| **TIDieR** | **INTERVENTION** | **CONTROL** |
| --- | --- | --- |
|  | **Davidson 2015** | |
| MATERIALS | Procedure materials: iPad Contraception provision: free LARCS, a month supply of contraceptive pill patch or ring or a depot medroxyprogesterone acetate injection | None |
| PROCEDURES | 1. Intervention video: The video intervention was comprised of 3 segments and delivered on an iPad. The first segment featured a health care provider delivering basic information about the 3 LARC methods (i.e., levonorgestrel IUD [LNG-IUD], copper IUD, and contraceptive implant). The second and third segments featured narrative comments from patients who had used a LARC following a surgical abortion. 2. Usual Care Including contraception and abortion counseling by clinic staff (during which time all contraceptive methods were discussed with the participant) and then underwent the abortion procedure". 3. Post - abortion survey: Following the abortion procedure, all participants completed a 5-item questionnaire that addressed their overall satisfaction with the contraceptive counseling and their perceived autonomy in their contraceptive decision making. | 1.Controlvideo: doctor discussing stress management  2. Usual Care: including contraception and abortion counseling by clinic staff (during which time all contraceptive methods were discussed with the participant) and then underwent the abortion procedure. 3. Survey post abortion: All participants completed a 5-item questionnaire that addressed their overall satisfaction with the contraceptive counseling and their perceived autonomy in their contraceptive decision making |
| WHO PROVIDED | Standard Care and Intervention: clinic staff" | Standard Care and intervention: clinic staff |
| HOW | Face to face | Face to face |
| WHERE | Free standing clinic | Free standing clinic |
| WHEN | Pre-abortion | Pre-abortion |
| HOW MUCH | Once | Once |
| TAILORING | Consistent with the TTM (The Prochaska’s transtheoretical model), video content was designed to facilitate LARC initiation by increasing women’s awareness of LARC, helping women weigh the pros and cons of LARC use and gain self-efficacy for using LARC in the postabortal period. | No detail |
| MODIFICATIONS | Unexpected protocol deviations, and thus, the decision was made to overrecruit 5 additional participants for the potential loss to follow up. | Unexpected protocol deviations, and thus, the decision was made to overrecruit 5 additional participants for the potential loss to follow up. |
| Adherence evaluation | No | No |
