# Supplementary material for: Peri-abortion contraceptive counseling: A systematic review of randomized controlled trials
Source: PLoS One. 2021 Dec 28;16(12):e0260794. doi: 10.1371/journal.pone.0260794 (PMC8714105; doi:10.1371/journal.pone.0260794)
Supplement: S12 Table — (DOCX) [file pone.0260794.s013.docx]

**S12 Table. Detail of the interventions received in Smith´s study.**

| **TIDieR** | **INTERVENTION** | **CONTROL** |
| --- | --- | --- |
|  | **Smith 2015** | |
| MATERIALS | Procedure materials: text messages | None |
| PROCEDURES | 1.- Standard care 2.- Six automated interactive voice messages at the time of their preference for 3 months. first message 11 week after abortion and every 2 weeks thereafter  3.- Additional message to the ones who selected oral or injectable contraceptive. | Standard care |
| WHO PROVIDED | Standard care and intervention: counsellor | Standard Care: Not specified |
| HOW | Voice messages and phone calls | No specified. |
| WHERE | Four Marie Stopes International clinics in Cambodia | Four Marie Stopes International clinics in Cambodia |
| WHEN | Post-abortion | Post-abortion |
| HOW MUCH | Six messages for 3 months | Once |
| TAILORING | Personalized. Participants who pressed 1 or who did not respond received a phone call from a counsellor. "The phone calls were intended to encourage contraceptive use by increasing the client’s capability of using contraception by: (i) providing individualized information on a range of contraceptive methods; (ii) increasing the participant’s opportunity to use contraception, for example, by informing her where she could access specific methods near her residence; and (iii) increasing motivation by reinforcing knowledge of the benefits of contraception. At the participant’s request, the counsellor would also discuss contraception with her husband or partner. Participants were also able to call the service and ask to speak to a counsellor. Those who chose to receive an oral or injectable contraceptive could opt to receive additional reminder messages appropriate to their method (ie. on when to start a new packet of pills or when to receive a new injection). The sixth and final voice message was similar but also reminded the participant that this was the last message they would receive." | Clinic phone number were provided for consults |
| MODIFICATIONS | No | No |
| Adherence evaluation | No | no |
