# Supplementary material for: Peri-abortion contraceptive counseling: A systematic review of randomized controlled trials
Source: PLoS One. 2021 Dec 28;16(12):e0260794. doi: 10.1371/journal.pone.0260794 (PMC8714105; doi:10.1371/journal.pone.0260794)
Supplement: S13 Table — (DOCX) [file pone.0260794.s014.docx]

**S13 Table. Detail of the interventions received in Olavarrieta´s study.**

| **TIDieR** | **INTERVENTION** | **CONTROL** |
| --- | --- | --- |
|  | **Olavarrieta 2015** | |
| MATERIALS | None | None |
| PROCEDURES | 1. All women received misoprostol, instructions for administration and contraceptive method counselling from their assigned provider. 2. The study followed the standard of care in counselling and providers offered a mix of different contraceptive methods. | 1. All women received misoprostol, instructions for administration and contraceptive method counselling from their assigned provider. 2. The study followed the standard of care in counselling and providers offered a mix of different contraceptive methods. |
| WHO PROVIDED | Nurse | Physician |
| HOW | No specified | No specified. |
| WHERE | Two Mexico City Ministry of Health abortion clinics and one hospital | Two Mexico City Ministry of Health abortion clinics and one hospital |
| WHEN | Post-abortion | Post-abortion |
| HOW MUCH | No detail | No detail |
| TAILORING | No detail | No detail |
| MODIFICATIONS | No | No |
| Adherence evaluation | No | No |
