# Supplementary material for: Peri-abortion contraceptive counseling: A systematic review of randomized controlled trials
Source: PLoS One. 2021 Dec 28;16(12):e0260794. doi: 10.1371/journal.pone.0260794 (PMC8714105; doi:10.1371/journal.pone.0260794)
Supplement: S14 Table — (DOCX) [file pone.0260794.s015.docx]

**S14 Table. Detail of the interventions received in Whitaker´s study.**

| **TIDieR** | **INTERVENTION** | **CONTROL** |
| --- | --- | --- |
|  | **Whitaker 2016** | |
| MATERIALS | Contraception provision: IUD and Hormonal contraceptive | Contraception provision: IUD and hormonal contraceptive |
| PROCEDURES | Motivational Interview (seven steps) and non standarized contraception counseling. The seven steps of the intervention were not designed to be a static outline, and counselors were free to move between the steps in a fluid manner but were instructed to include all seven steps: (1) establish rapport, (2) set the agenda, (3) discuss prior contraception use, (4) ask permission to give educational information about contraceptive methods, (5) assess importance, confidence and readiness to use contraception, (6) continued discussion of very effective contraception, and (7) wrap up. | Non standarize contraceptive counselling |
| WHO PROVIDED | Standard care: Clinic physician Intervention: Seven step motivational Interview given once, provided by a physician or social worker given once and duration not specified | Standard Care: clinic physician |
| HOW | Not specified | Not specified |
| WHERE | Urban academic center | Urban academic clinic |
| WHEN | Not specified | Not specified |
| HOW MUCH | Once | Once |
| TAILORING | Seven step motivational Interview given once. | Not specified |
| MODIFICATIONS | No | No |
| Adherence evaluation | No | No |
