# Supplementary material for: Peri-abortion contraceptive counseling: A systematic review of randomized controlled trials
Source: PLoS One. 2021 Dec 28;16(12):e0260794. doi: 10.1371/journal.pone.0260794 (PMC8714105; doi:10.1371/journal.pone.0260794)
Supplement: S15 Table — (DOCX) [file pone.0260794.s016.docx]

**S15 Table. Detail of the interventions received in Makenzius´s study.**

| **TIDieR** | **INTERVENTION** | **CONTROL** |
| --- | --- | --- |
|  | **Makenzius 2017** | |
| MATERIALS | None | None |
| PROCEDURES | Before discharge offered contraceptive counselling and provided with a contraceptive method if they wanted and given follow up after 7-10d. | Before discharge offered contraceptive counselling and provided with a contraceptive method if they wanted and given follow up after 7-10d. |
| WHO PROVIDED | Midwife. | Physician |
| HOW | Not specified | Not specified |
| WHERE | Jaramogi Oginga Odinga Teaching and Referral Hospital (JOOTRH) and Kisumu County Hospital (KCH) in Kisumu County, western Kenya | Jaramogi Oginga Odinga Teaching and Referral Hospital (JOOTRH) and Kisumu County Hospital (KCH) in Kisumu County, western Kenya |
| WHEN | Post-abortion | Post-abortion |
| HOW MUCH | Once | Once |
| TAILORING | None | None |
| MODIFICATIONS | no | No |
| Adherence evaluation | No | No |
