# Supplementary material for: Peri-abortion contraceptive counseling: A systematic review of randomized controlled trials
Source: PLoS One. 2021 Dec 28;16(12):e0260794. doi: 10.1371/journal.pone.0260794 (PMC8714105; doi:10.1371/journal.pone.0260794)
Supplement: Attachment — Submitted filename: PLOS ONE SYSTEMATIC REVIEWE, COMMENTS _GOA.docx [file pone.0260794.s018.docx]

GENERAL COMMENTS

This is a relevant systematic review which is intended to provide robust evidence regarding the effectiveness of peri-abortion contraceptive counseling in several settings towards reduction of the incidences of repeat abortions.

ABSTRACT

Comment 1: The time frame for the studies included in the systematic review is conspicuously missing. This should be reflected not in the abstract.

Comment 2: Also include the databases searched by the authors ad whether there were language restrictions in the search.

Conclusion

Comment 3: The authors stated in the conclusion “There is no evidence that involvement of different professionals influences contraceptive use”. It is not clear how this was arrived at since only two studies out of 11 compared influence of the involvement of different professionals in counselling. Rather due to the paucity of RCT that compared the two approaches, there is limited evidence that involvement of different professionals influences contraceptive use.

Key messages

Comment 4: The key messages from this study has not been well highlighted in this section. The following statements should be deleted from this section during revision:

•We conducted a systematic review and meta-analysis of 11 RCTs to determine the effect of peri-abortion contraceptive counseling.

• We evaluated the effect of contraceptive counseling on peri-abortion contraceptive use to assess the contribution of this strategy to reducing future pregnancies.

Replace the above with information that highlights key messages like whether this was the first RCT on the subject matter, uniqueness of study, findings, conclusions, and recommendations:

Introduction

Page 4, lines 2-3: Globally, abortion is a complex situation for people with uterus (cis, trans and non-binary people). [2].

Comment 5: This statement may not be relevant to the context. Please delete

Page 4, Lines 14-17: Therefore, we performed a systematic review to summarize the available data on randomized controlled trials (RCTs) effects of peri-abortion contraceptive counseling intervention on contraceptive use and obstetric outcomes.

Comment 6: Paraphrase to: Therefore, we performed a systematic review to summarize the available data from randomized controlled trials (RCTs) that evaluated the effects of peri-abortion contraceptive counseling intervention on contraceptive use and obstetric outcomes

Methods

Page 4, line 24, Page 5 lines 1-2: and enhanced the new strategy considered for the trial) and were published as original papers in scientific journals

Comment 7: and enhanced counselling as the new strategy considered for the trial) and were published as original papers in scientific journals.

Information sources and search strategy

Page 5, lines 6-8: We performed a literature search of four sources: PubMed, Central Cochrane Library (CENTRAL), Scopus, and Google Scholar (for this, we only assessed the first 100 results).

Comment 8: It is not clear why only the first 100 results from google scholar was assessed. This should be elucidated upon.

Outcomes of interest

Comment 9: The authors have listed various definition of terms considered for this study but has not referenced these considerations for objectivity in assessment of these studies in relation to widely accepted definitions. This should be included in the revision.

Results

Page 11, lines 1-2: In addition, the proportion of women leaving the facility with at least one contraceptive method was similar in the “nurse group” than in the “physician group”

Comment 10: Change to: In addition, the proportion of women leaving the facility with at least one contraceptive method was similar in both groups

Discussion

This section has been well written and exhaustive

Page 11, line 18: which include six RCTs

Comment 11: which included six RCTs

Limitations

The authors have highlighted the main limitations to this study and interpretation of results.

Strength

However, to date, this is the most comprehensive systematic review that has assessed the effects of peri-abortion contraceptive counseling, summarizing information that may be useful to informed decision-making.

Comment 12: This message should be adapted as part of the key message from this study.

References

The journal names should be written as they are abbreviated in the list of index medicus for all the references quoted.
